# Lattice matching enables construction of CaS@NaYF_4_ heterostructure with synergistically enhanced water resistance and luminescence for antibiotic detection

**DOI:** 10.1007/s00604-024-06568-x

**Published:** 2024-07-26

**Authors:** Yao Wang, Huadong Chen, Tonghan Zhao, Jing Wang, Yihan Wu, Jinliang Liu, Yong Zhang, Xiaohui Zhu

**Affiliations:** 1 School of Environmental and Chemical Engineering, Shanghai University, Shanghai, 200444 China; 2 Department of Biomedical Engineering, City University of Hong Kong, Hong Kong SAR, 999077 China

**Keywords:** Nanosized CaS, Water resistance, Lattice matching, FRET, Antibiotic detection

## Abstract

Rare earth (RE)-doped CaS phosphors have been widely used as light-emitting components in various fields. Nevertheless, the application of nanosized CaS particles is still significantly limited by their poor water resistance and weak luminescence. Herein, a lattice-matching strategy is developed by growing an inert shell of cubic NaYF_4_ phase on the CaS luminescent core. Due to their similarity in crystal structure, a uniform core–shell heterostructure (CaS:Ce^3+^@NaYF_4_) can be obtained, which effectively protects the CaS:Ce^3+^ core from degradation in aqueous environment and enhances its luminescence intensity. As a proof of concept, a label-free aptasensor is further constructed by combining core–shell CaS:Ce^3+^@NaYF_4_ and Au nanoparticles (AuNPs) for the ultrasensitive detection of kanamycin antibiotics. Based on the efficient FRET process, the detection linear range of kanamycin spans from 100 to 1000 nM with a detection limit of 7.8 nM. Besides, the aptasensor shows excellent selectivity towards kanamycin antibiotics, and has been successfully applied to the detection of kanamycin spiked in tap water and milk samples, demonstrating its high potential for sensing applications.

**Supplementary Information:**

The online version contains supplementary material available at 10.1007/s00604-024-06568-x.

## Introduction

Rare earth (RE) metal refers to a group of 17 chemical elements, including lanthanides (La–Lu) and transition metals of scandium (Sc) and yttrium (Y) [1–3]. Due to its distinctive electron configurations and rich energy levels, rare earth doping has been considered as a routine strategy to explore optical materials with tunable emission profiles, low photobleaching, and high color purity [4, 5]. Among them, RE-doped alkaline-earth sulfide,like CaS, has attracted increasing attentions. Various color outputs can be accomplished through doping RE ions in CaS, such as CaS:Eu^2+^ for red light [6, 7], CaS:Bi^2+^ for blue light [8, 9], and CaS:Ce^3+^ for green light [10–12]. Due to its intense luminescence, high quantum yield and ease of color tunability, RE-doped CaS has been widely used in lighting, sensing, and anti-counterfeiting [13–15].

Traditional synthesis of CaS phosphors always requires high-temperature solid-state reactions [16–18], which, however, tend to produce particles with large size (~ μm) and non-uniform morphology. Therefore, this presents significant challenges for surface modification, especially when applied at the nanoscale. Recently, nano-sized CaS particle, ranging from 20 to 30 nm, was synthesized using high-temperature co-precipitation method, demonstrating great efficiency for xanthine detection [19]. Despite these achievements, there are still several critical challenges to address regarding the application of nanoscale CaS particles. Firstly, the luminescence quantum yield of nanosized CaS is much smaller than its bulk or microsized counterparts. This is mainly because nanosized particle generally has a large surface-to-volume ratio, which leads to significant quenching effects caused by the abundant luminescence quenchers on the surface, such as defects and high-energy vibrational groups (e.g., ligands and solvent molecules [20, 21]. Besides, CaS-based phosphors are not stable in aqueous media as they are prone to hydrolysis [22], which further limits their sensing and imaging applications.

Kanamycin, an alkaline aminoglycoside antibiotic, is widely used for treating bacterial infections and is prevalent as a veterinary drug in livestock and aquaculture [23]. However, excessive use leads to KAN accumulation in animal-derived foods, posing health risks like nephrotoxicity, ototoxicity, and allergies upon consumption [24]. Therefore, it is imperative to establish an accurate method for detecting KAN. Traditional methods for KAN detection include enzyme-linked immunosorbent assay (ELISA) [25, 26], high performance liquid chromatography (HPLC) [27, 28], and capillary electrophoresis (CE) [29, 30], which are known for their precision and high detection accuracy. Nevertheless, these techniques require expensive equipment and the detection process is complicated and time-consuming. Therefore, it is of great significance to establish a rapid method for KAN detection that is easy to operate and highly sensitive.

Fluorescence resonance energy transfer (FRET) is a phenomenon that characterizes the energy transfer between two photosensitive molecules, i.e., energy donor and energy acceptor [31]. It requires that the spatial distance between the energy donor and the energy acceptor should be less than 10 nm [32]. Besides, the emission spectrum of the energy donor should overlap with the absorption spectrum of the energy acceptor [32]. Because of its high sensitivity, FRET emerges as a powerful technique in the realm of sensing and detection. By introducing the target substance, the transfer process between the energy donor and acceptor can be modulated, which then alters the intensity or lifetime of fluorescence [33]. This, in turn, can be used as the fingerprint for quantitative detection of targeted analytes.

Aptamer is a short and single-stranded DNA or RNA molecule, which is generally produced from a process, known as SELEX (systematic evolution of ligands by exponential enrichment) [34]. Because it can also recognize and bind to target molecules, aptamer has been considered as an alluring alternative to antibodies. Particularly, due to its high affinity and specificity, ease of modification and high thermal stability [35], aptamers have provided sensitive and versatile solutions to detect diverse target molecules in the biological and environmental samples. In recent years, various types of aptasensors based on different signal transducers, such as electrochemistry [36], fluorescence [37], and colorimetry [38], have been explored and successfully used for detection of ochratoxin A, oligosaccharide, blood biomarker, etc. Despite of these achievements, the reported aptasensors still suffer from limited sensitivity and specificity.

**Scheme 1:**
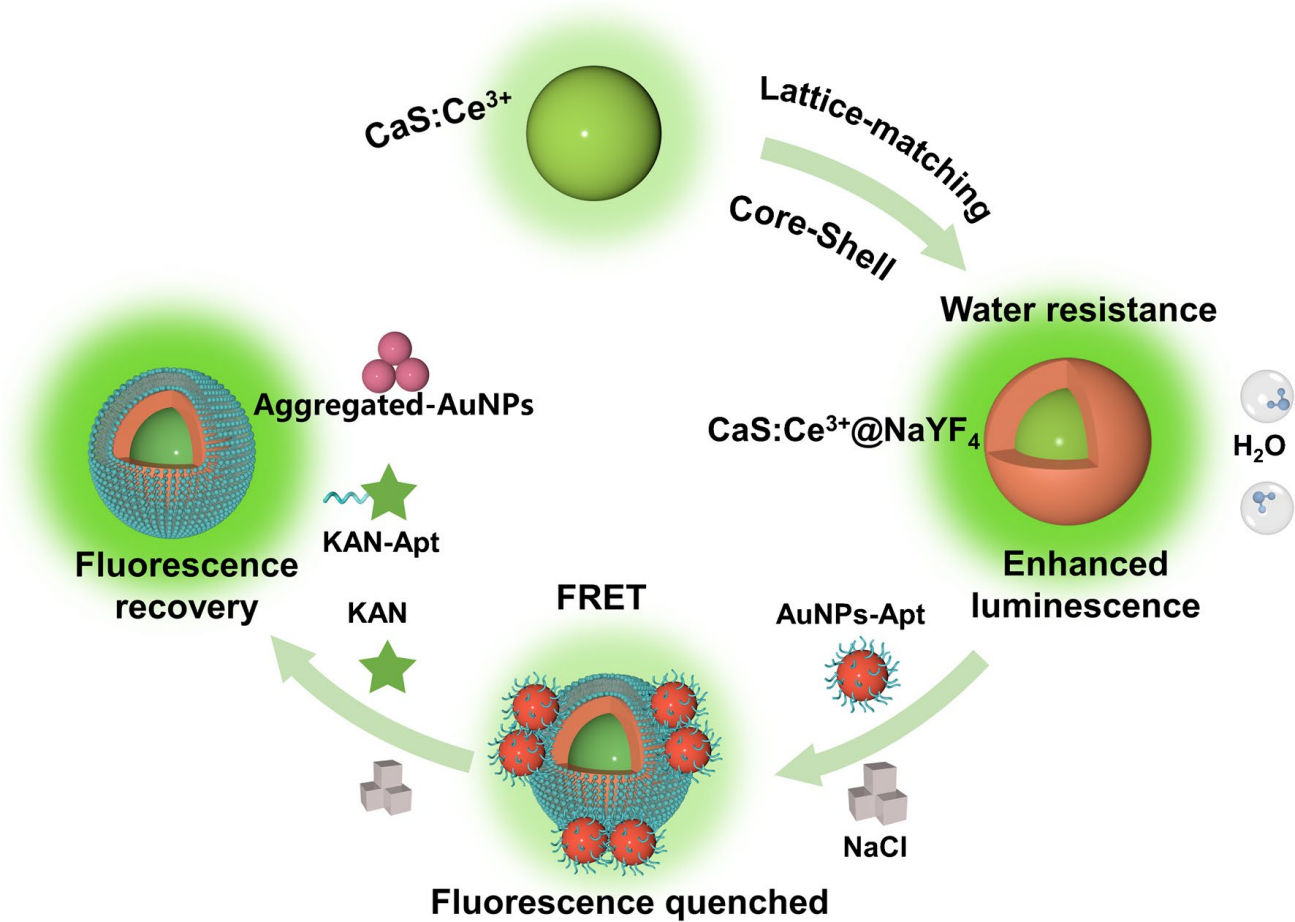
Schematic of a label-free aptasensor based on CaS:Ce@NaYF_4_ and Au nanoparticles for kanamycin detection

It has been reported that CaS belongs to the space group of *Fm* $\overline{3 }$ *m* with a typical cubic crystal structure (a = b = c = 5.68 Å) [39]. Firstly, this study explores a lattice-matching strategy by encapsulating the CaS core with an optically inactive NaYF_4_ shell. The cubic NaYF_4_ phase is specifically selected as the shell material because it has the similar lattice parameter to CaS with a lattice misfit of only about 3.6% [40]. With the presence of NaYF_4_ shell, the water-resistance capability of CaS core is improved by 5 times and luminescence intensity by 1.8 times. Furthermore, this study develops a label-free aptasensor based on core–shell CaS:Ce^3+^@NaYF_4_ and Au nanoparticles (AuNPs) for ultrasensitive detection of Kanamycin (KAN), where CaS:Ce^3+^@NaYF_4_ acts as an energy donor and AuNPs as an energy acceptor **(**Scheme 1**)**. Without KAN, AuNPs bound to the aptamer exhibit strong electrostatic repulsion, ensuring stable dispersion in salt solution. With KAN present, however, the aptamer specifically binds to KAN, causing AuNPs to aggregate and reducing absorption intensity. Consequently, the FRET efficiency is affected, with the fluorescence of CaS:Ce^3+^@NaYF_4_ being quenched first and then recovered upon the presence of KAN. In other words, the concentration of KAN can be probed based on the degree of fluorescence recovery of CaS:Ce^3+^@NaYF_4_. Our results indicate that the as-developed aptasensor demonstrates a wide detection linear range of KAN from 100 to 1000 nM, with a detection limit of 7.8 nM. Besides, it shows excellent selectivity towards KAN in tap water and milk samples, confirming its great potential for sensing applications.

## Results and discussion

### Characterization of CaS:Ce^3+^ and CaS:Ce^3+^@ NaYF_4_

**Fig. 1:**
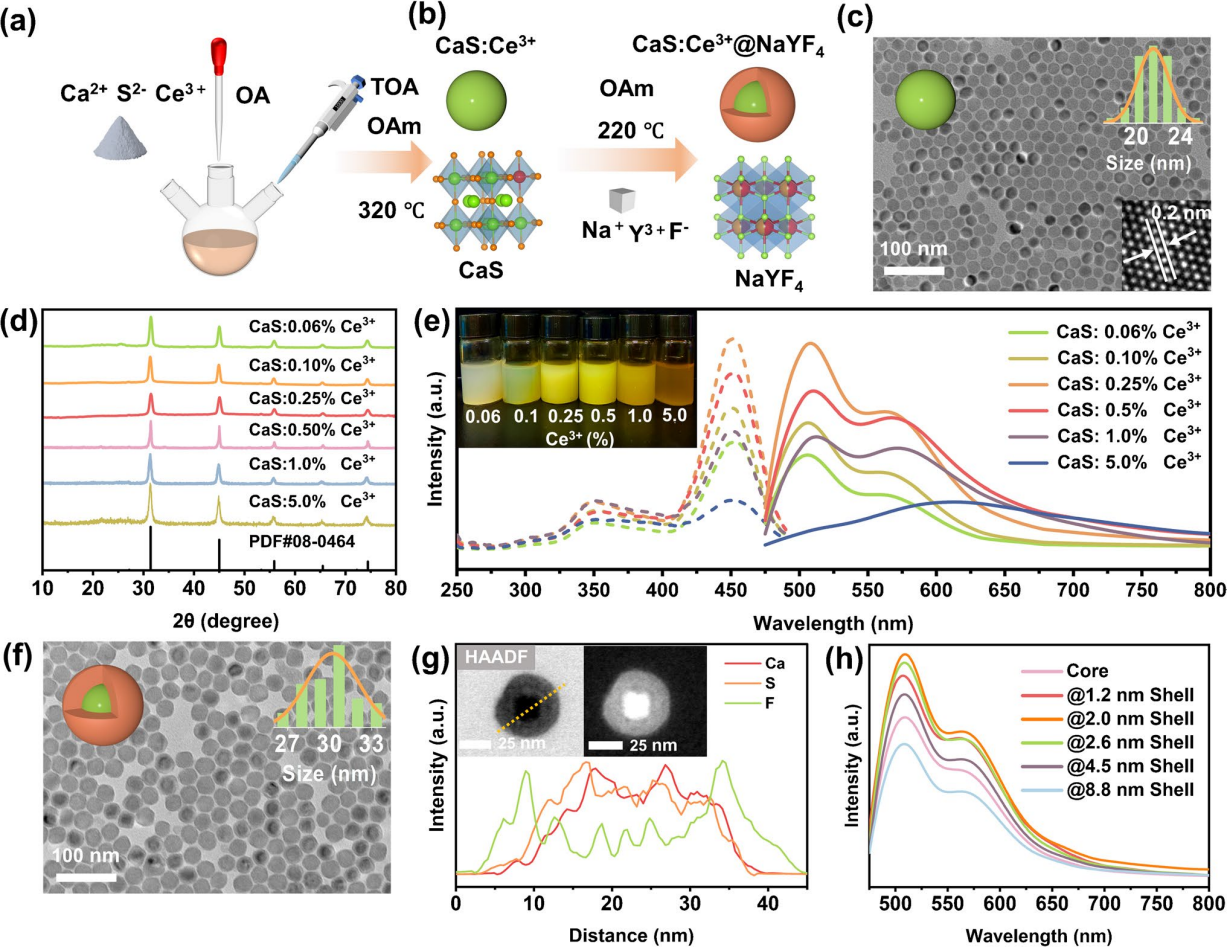
**a** Schematic for the synthesis of CaS:Ce^3+^ nanoparticles; (**b**) Illustration of the synthesis of core–shell CaS:Ce^3+^@NaYF_4_ nanoparticles (Top), Cubic NaYF_4_ is selected as the shell material because it has a similar crystal structure to CaS (bottom); (**c**) TEM image and corresponding size distribution of CaS:0.25% Ce^3+^ nanoparticles, The inset shows the lattice spacing of CaS:Ce^3+^; (**d**) XRD pattern of synthesized CaS:x% Ce^3+^ nanoparticles; (**e**) Left: luminescence excitation spectra of CaS:x% Ce^3+^ solution (0.1 mol/L) obtained by monitoring emission at 515 nm, Right: luminescence emission spectra of CaS:x% Ce^3+^ upon excitation by 450 nm light, Inset: photograph of the CaS:x% Ce^3+^ nanoparticles in cyclohexane dispersion under ambient light; (**f**) TEM image and corresponding size distribution of CaS:Ce^3+^@NaYF_4_ nanoparticles; (**g**) Top: high-angle annular dark-field scanning transmission electron microscopy (HAADF-STEM) image of a single CaS:Ce^3+^@NaYF_4_ nanoparticle, Bottom: EDX-line scan spectra across the CaS:Ce^3+^@NaYF_4_ nanoparticle; (**h**) Luminescence emission spectra of CaS:Ce^3+^@NaYF_4_ nanoparticles with different thicknesses of NaYF_4_ shell

Herein, Ce^3+^ doped CaS (CaS:Ce^3+^) nanoparticles were first synthesized using a modified high-temperature co-precipitation method **(**Fig. 1a**)** [19]. Then, another layer of cubic NaYF_4_ shell with a similar crystal structure was further coated on the CaS:Ce^3+^ core **(**Fig. 1b**)**. Figure 1c presents the transmission electron microscopy (TEM) image of as-synthesized CaS:Ce^3+^ nanoparticles with uniform morphology. The mean size of the nanoparticles was calculated to be 21.3 ± 1.5 nm by randomly selecting 200 nanoparticles from the TEM image. The low polydispersity index (PDI) of 0.15 ± 0.02 obtained from dynamic light scattering (DLS) further demonstrated that the particles are monodisperse in solution (Fig. S1). The high-resolution TEM (HRTEM) image (the inset in Fig. 1c) further reveals the lattice spacing is about 0.20 nm, which corresponds to the (220) plane of cubic CaS. As the major luminescent center, the Ce^3+^ dopant plays a vital role in the luminescence performance of CaS:Ce^3+^. Therefore, CaS:x% Ce^3+^ (x = 0.06, 0.1, 0.25, 0.5, 1, and 5) nanoparticles with different doping concentrations of Ce^3+^ were synthesized. As shown in Fig. S2, the mean particle size gradually decreases from 28.6 ± 2.0 nm to 18.6 ± 1.4 nm with increasing Ce^3+^ doping concentration. The size reduction is attributed to alterations in the crystal surface charge caused by Ce^3+^ doping because it can decrease the diffusion rate of Ce^3+^ into the host lattice and thus restrain crystal growth [41]. Additionally, the X-ray diffraction (XRD) pattern **(**Fig. 1d**)** shows that all the synthesized CaS:x% Ce^3+^ can be accurately labeled to cubic CaS (JCPDS No. 008–0464), confirming the successful synthesis of pure CaS nanocrystals.

Figure 1e compares the excitation and emission spectra of CaS nanoparticles with different Ce^3+^ doping concentrations. Clearly, all the excitation spectra show two prominent peaks centered at 350 nm and 450 nm by monitoring the emission at 515 nm, which corresponds to the 4*f* → 5*d* (T_2g_) transition of Ce^3+^. Under excitation at 450 nm, the CaS:Ce^3+^ nanoparticles exhibit two broad emissions at around 515 nm and 565 nm, which can be attributed to transitions of 5*d* → 4*f* (^2^F_7/2_ and ^2^F_7/2_) in Ce^3+^ [42]. Since Ce^3^⁺ ions serve as the luminescence centers for CaS:Ce^3+^ nanoparticles, the luminescent emission initially increases with Ce^3^⁺ concentration and reaches maximum at doping concentration of 0.25% (Fig. S3a). However, with further increasing Ce^3^⁺ concentration, the distance among Ce^3+^ ions is also reduced. Such proximity leads to stronger interactions and energy transfer among Ce^3+^ dopants, which significantly raises the possibility of encountering luminescence quenching sites. As a result, non-radiative transitions may occur through the concentration quenching process, which diminishes the luminescence emission intensity. Therefore, when the Ce^3^⁺ doping concentration exceeds 0.25%, a reduction in luminescence intensity is observed. As a result, the optimal Ce^3+^ doping concentration is determined to be 0.25%.

Besides, it can be observed that there is a redshift of the emission spectrum (from 515 to 565 nm) upon Ce^3+^ doping. As shown in the inset of Fig. 1e, with the increase of Ce^3+^ doping concentration, the color of the synthesized CaS:x% Ce^3+^ nanoparticles in the solution gradually changes from light green to brownish yellow. It has been reported that there are two major types of Ce^3+^ sites in the CaS: Ce^3+^ nanoparticles (Fig. S3b) [43]. One is the regular Ce^3+^ cation site within the bulk CaS nanocrystal (regular Ce^3+^), which emits green emission at 515 nm. The other type is located at perturbed surface sites (perturbed Ce^3+^), which mainly produces emission at 565 nm. Because these perturbed Ce^3+^ ions are more exposed to surface quenchers compared to regular Ce^3+^, their emission at 515 nm is accordingly more quenched, leading to a yellow-dominated emission profile. Upon Ce^3+^ doping, there are some factors contributing to the redshift of emission spectrum from 515 to 565 nm. One factor is the initiation of energy hopping among Ce^3^⁺ ions at high doping levels [44], which facilitates energy transfer from the bulk Ce^3^⁺ ions to surface quenchers, leading to a noticeable decrease in the emission at 515 nm. Another factor is the creation of Ce^3+^-related color centers (dark Ce^3+^) on the nanoparticle surface [20]. These centers are caused by the aliovalent doping, which were thought to be associated with the formation of interstitial S^2−^ for charge compensation. The S-rich environment further positions Ce^3+^ absorption via 4*f* → 5*d* transition in the green region [20], selectively quenching the emission at 515 nm.

Then, CaS:0.25% Ce^3+^ nanoparticle was selected as the seed and coated with a layer of NaYF_4_ inert shell. Specifically, the trifluoroacetate was first dissolved in a certain amount of oleylamine to prepare the precursor solution. Subsequently, a portion of pre-synthesized CaS seeds was added to the precursor solution. After removing the cyclohexane under argon protection, the mixture was heated to 220 ℃ for two hours. Detailed synthesis steps are provided in the supporting information. As can be seen in Fig. 1f, the synthesized nanoparticles exhibit a larger size (30.2 ± 1.7 nm) than the CaS core (Fig. 1c). Both the TEM image and DLS analysis (Fig. S4) reveal that the synthesized nanoparticles maintain a homogeneous morphology (PDI = 0.19 ± 0.03). Furthermore, Fig. 1g displays a high-angle annular dark-field scanning transmission electron microscopy (HAADF-STEM) image of a single CaS:Ce^3+^@NaYF_4_ nanoparticle, which clearly demonstrates a core–shell morphology (bright-dark contrast). The EDX-line scan spectra also indicate a higher Ca^2+^ and S^2−^ concentration in the center and a higher F^−^ concentration in the peripheral region of the particle, which is consistent with the expected elemental distribution for the core–shell CaS:Ce^3+^@NaYF_4_ structure.

Next, the influence of the NaYF_4_ inert shell on the luminescent intensity of CaS:Ce^3+^@NaYF_4_ was investigated. By varying the addition of shell precursor, a series of CaS:Ce^3+^@NaYF_4_ nanoparticles with different shell thicknesses (1.2 nm ~ 8.8 nm) were obtained. The TEM images in Fig. S5 reveal that all the synthesized nanoparticles maintain uniform morphology and gradually increase in size. As can be seen in Fig. 1h, the emission intensity at 515 nm initially enhances as the thickness of the NaYF_4_ shell increases, reaching a maximum enhancement of 1.8 times at a shell thickness of 2.0 nm. In addition, the photoluminescence quantum yield (PLQY) of the nanoparticle was increased from 31.4% to 40.8% after coating the inert layer (Supporting Table S1). These results clearly indicate that a certain thickness of NaYF_4_ inert layer can effectively passivate surface defects and suppress non-radiative recombination, thereby enhancing luminescence efficiency. However, with further increase in the shell thickness, the luminescence intensity gradually diminishes, possibly due to some detrimental energy lost during the energy transfer in the excessively thick shell. As reported by Xu et al. [45], thicker outer shells may introduce more internal defects that partially dissipate the excitation energy. Moreover, a thicker shell enlarges the distance between the luminescent core and Au nanoparticles, potentially deteriorating the energy transfer between donors and acceptors and decreasing FRET efficiency. Consequently, the optimal NaYF₄ shell thickness is determined to be about 2.0 nm.

**Fig. 2:**
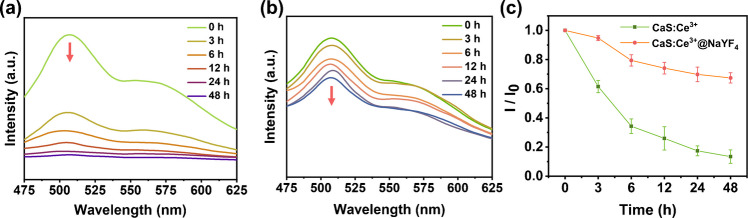
**a **Time-dependent fluorescence emission spectra of CaS:Ce^3+^ in aqueous solution (0.1 mol/L); (**b**) Time-dependent fluorescence emission spectra of CaS:Ce^3+^@NaYF_4_ in aqueous solution; (**c**) Time-dependent fluorescence intensity curve of CaS:Ce^3+^ and CaS:Ce^3+^@NaYF_4_ (I_0_: Fluorescence intensity at 515 nm at 0 h, I: Fluorescence intensity at 515 nm at different time points)

It is well known that pure CaS is not stable in aqueous solution because it can react with water and produce hydrogen sulfide (H_2_S) gas [22], which severely limits their application as a fluorescent probe. However, we propose that the inert NaYF_4_ shell can effectively isolate the CaS core from contacting water, therefore enhancing its water-resistance ability. To verify this hypothesis, pure CaS:Ce^3+^ and CaS:Ce^3+^@NaYF_4_ nanoparticles were dissolved in water and their luminescent spectra were recorded over time, which were shown in Fig. 2a and Fig. 2b. For a better comparison, Fig. 2c compares the decay of luminescence intensity at around 515 nm for CaS:Ce^3+^ (green) and CaS:Ce^3+^@NaYF_4_ (red) over 48 h. Due to the reaction with water, the degradation of the pure CaS core leads to a noticeable decline in emission intensity. Specifically, the luminescence intensity for CaS:Ce^3+^ core drops to just 13% of its initial intensity. In contrast, the luminescence intensity of CaS:Ce^3+^@NaYF_4_ still remains about 68% of the original intensity, which is over 5.2 times greater than that of the pure CaS:Ce^3+^ core. Thus, it can be concluded NaYF_4_ layer can offer protective benefits to the CaS nanoparticles by partially protecting them from degradation in aqueous environments.

### Construction of the FRET aptasensor

To further enhance the solubility of CaS:Ce^3+^@NaYF_4_ in water, a layer of amphiphilic phospholipid (lipo, DSPE-PEG2000) was then coated, which was able to enhance the stability of nanoparticles in solution. Successful coating of phospholipid was verified by Fourier-transform infrared spectroscopy (FTIR). As shown in Fig. S6a, the peaks at 2932 cm^−1^ and 2861 cm^−1^ of CaS:Ce^3+^@NaYF_4_ correspond to the asymmetric and symmetric stretching vibrations of the methylene group (-CH_2_-) in the oleic acid pre-existing on the particle surface. For the DSPE-PEG2000-coated CaS:Ce^3+^@NaYF_4_ nanoparticle (CaS:Ce^3+^@NaYF_4_@lipo), the appearance of new peaks at 1576 cm^−1^ and 1627 cm^−1^ are attributed to the amide bonds of the phospholipid molecules, indicating that DSPE-PEG2000 phospholipid was successfully coated on the surface of CaS:Ce^3+^@NaYF_4_ nanoparticles [19]. In addition, the DLS result (Fig. S6b) shows that the DLS size also increases from 36.8 ± 1.3 nm to 61.4 ± 1.7 nm, further confirming the coating of the DSPE-PEG2000. Additionally, the TEM image revealed that the wide distribution of CaS:Ce^3+^@NaYF_4_@lipo nanoparticles may be due to slight aggregation caused by the hydrophobic interaction of the phospholipids (Fig. S6c).

**Fig. 3:**
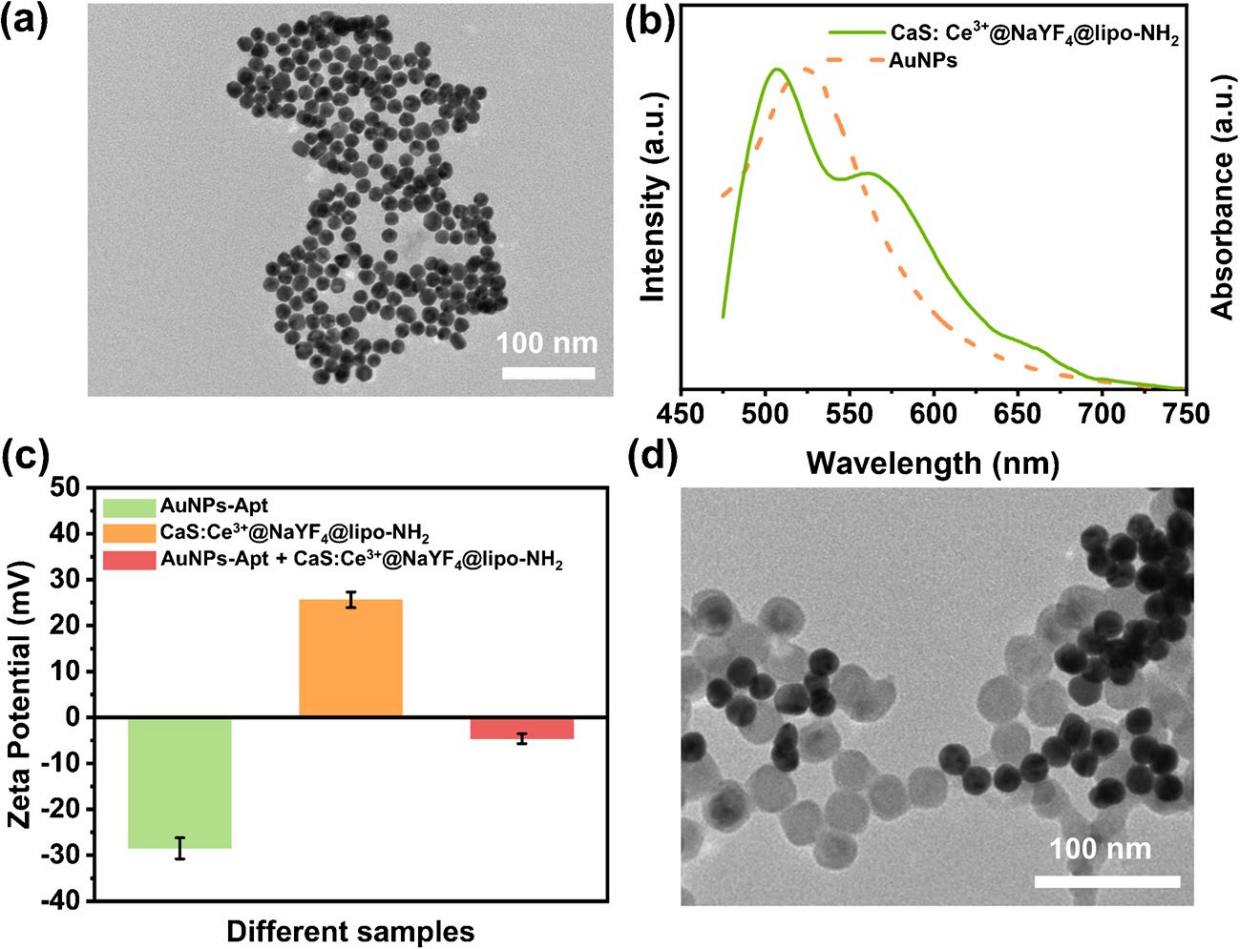
**a** TEM image of AuNPs; **b** Luminescence emission spectrum of CaS:Ce^3+^@NaYF_4_@lipo-NH_2_ solution (0.1 mol/L) and UV–Vis absorption spectrum of 17.2 nm AuNPs; **c** Zeta potentials of AuNPs-Apt, CaS:Ce^3+^@NaYF_4_@lipo and AuNPs-Apt + CaS:Ce^3+^@NaYF_4_@lipo-NH_2_; **d** TEM image of combination of CaS:Ce^3+^@NaYF_4_@lipo-NH_2_ with AuNPs-Apt due to electrostatic attraction

Based on successful surface modification, a FRET-based CaS:Ce^3+^@NaYF_4_-AuNPs (Au nanoparticles) nanosensor can be constructed, where CaS:Ce^3+^@NaYF_4_ as the energy donor and AuNPs as the energy acceptor. Firstly, a traditional reduction method [46] was used to synthesize AuNPs with a uniform morphology and size of 17.2 ± 1.1 nm (Fig. 3a). Notably, by adjusting the amount of sodium citrate added, we synthesized AuNPs with different sizes (Fig. S7). Meanwhile, with the increase in the size of AuNPs, the absorption peak exhibits a blueshift (Fig. S8a) and the color in aqueous solution also changes from deep purple to wine-red (Fig. S8b). This occurs because the size of AuNPs determines the frequency of their surface plasmon resonance, affecting their light absorption at specific wavelengths and resulting in changes in absorption spectra and solution color [47]. Figure 3b presents the absorption spectrum of as-synthesized AuNPs of 17.2 ± 1.1 nm, which shows a broad absorption peak at 520 nm. Therefore, an efficient energy transfer between CaS:Ce^3+^@NaYF_4_ and AuNPs is anticipated because there is an excellent overlap between the emission of CaS:Ce^3+^@NaYF_4_ and the absorption of AuNPs. For the detection purpose, the aptamer, a short nucleic acid chain, was then conjugated to the AuNPs (AuNPs-Apt) through coordination bonds formed between nitrogen atoms in the aptamer and gold atoms in the AuNPs [48]. The hydrodynamic diameter was increased after conjugation (Fig. S9a), indicating successful interaction between aptamers and AuNPs. In addition, it is observed that the modification with aptamers does not impact the absorption intensity of AuNPs (Fig. S9b).

Then, the surface of CaS:Ce^3+^@NaYF_4_@lipo nanoparticles were modified by NH_2_ groups to make them positively charged. Besides, the modified CaS:Ce^3+^@NaYF_4_@lipo-NH_2_ nanoparticles still maintain a strong fluorescence intensity (Fig. S9c). Figure 3c shows the zeta potential of AuNPs-Apt is -28 ± 1.7 mV while that of CaS:Ce^3+^@NaYF_4_@lipo-NH_2_ is + 25.6 ± 1.3 mV. When AuNPs-Apt is introduced into CaS:Ce^3+^@NaYF_4_@lipo-NH_2_, the zeta potential becomes -4.6 ± 0.7 mV. The results indicate that AuNPs-Apt can be well combined with CaS:Ce^3+^@NaYF_4_@lipo-NH_2_ nanoparticles due to the electrostatic attraction (Fig. 3d). This is because the positively charged CaS:Ce^3+^@NaYF_4_@lipo-NH_2_ becomes electrically neutral after adsorption with the negatively charged AuNPs-Apt, resulting in a decrease in the zeta potential. The reduction in repulsive forces among the adsorbed nanoparticles then causes aggregation. To verify the existence of energy transfer between CaS:Ce^3+^@NaYF_4_@lipo-NH_2_ and AuNPs-Apt, we measured the fluorescence decay lifetime of CaS:Ce^3+^@NaYF_4_@lipo-NH_2_ nanoparticles at 515 nm before and after the addition of AuNPs (Fig. S10). It can be obtained that the lifetime of Ce^3+^ ion emission at 515 nm was significantly shortened after the addition of AuNPs-Apt, confirming the energy transfer between the CaS:Ce^3+^@NaYF_4_@lipo-NH_2_ and AuNPs-Apt.

### Detection strategy of the FRET aptasensor for KAN

**Fig. 4:**
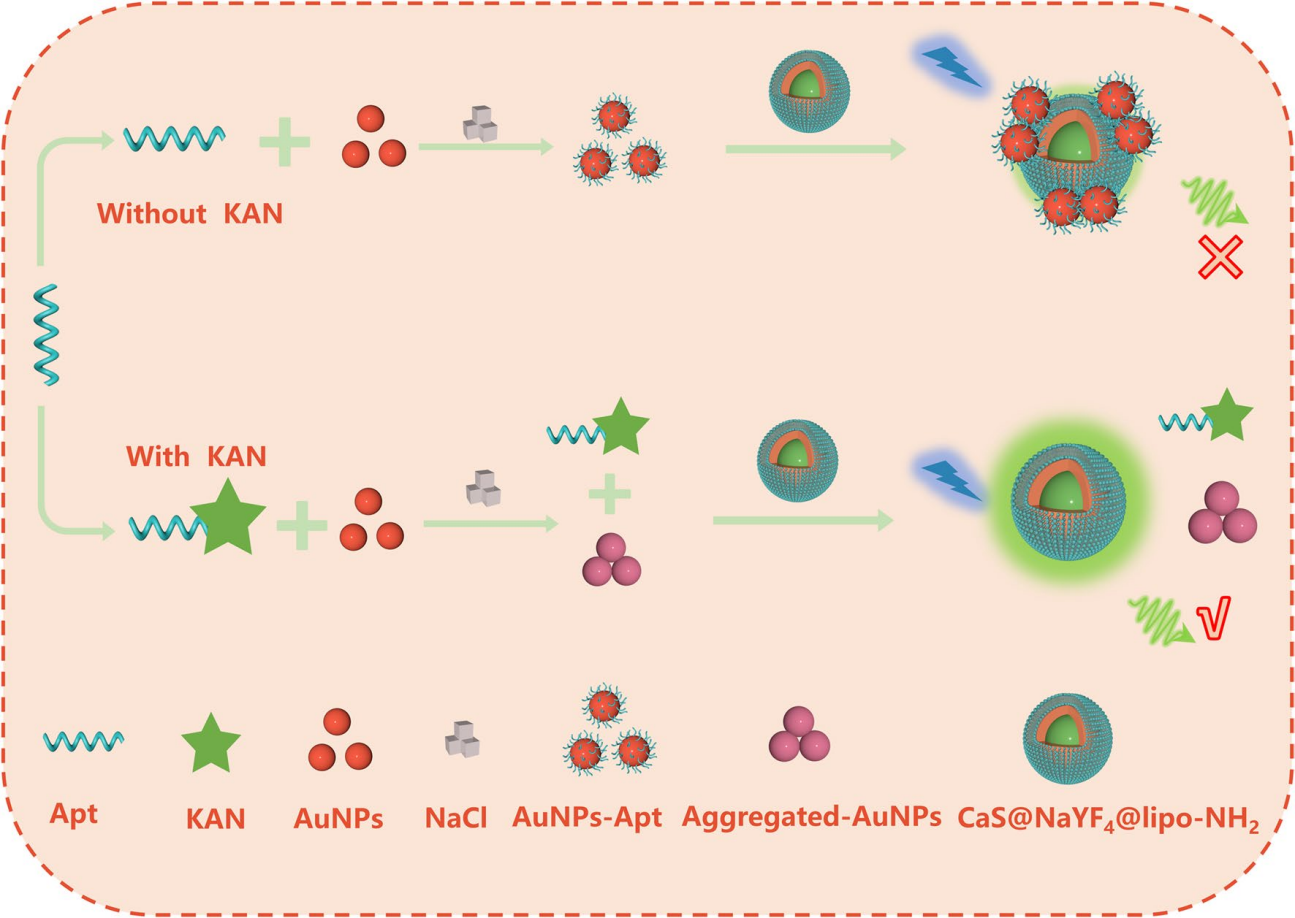
Schematic illustration of proposed aptasensor for KAN detection

**Fig. 5:**
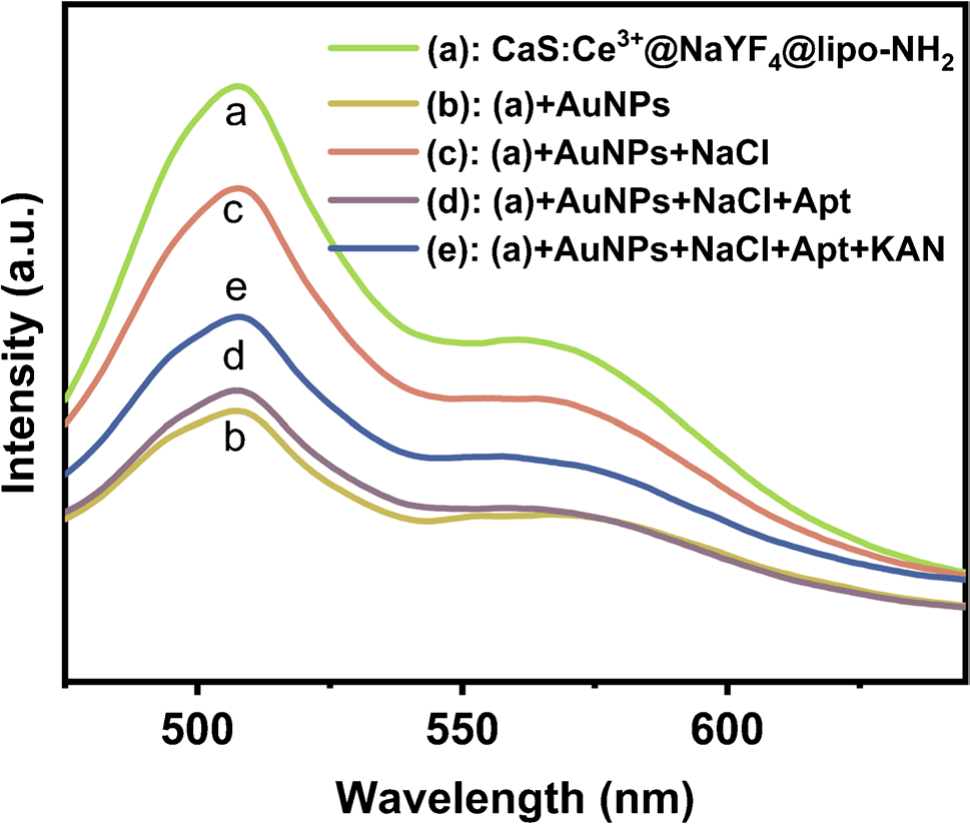
Fluorescence emission spectra (ex@450 nm) of CaS:Ce^3+^@NaYF_4_@lipo-NH_2_ solution (0.1 mol/L) with various components

The mechanism of KAN detection via FRET-based aptasensor based on CaS:Ce^3+^@NaYF_4_@lipo-NH_2_ and AuNPs-Apt is illustrated in Fig. 4. As shown in Fig. 5, the group of CaS:Ce^3+^@NaYF_4_@lipo-NH_2_ (line a) exhibits the highest fluorescent emission intensity at 515 nm among all the samples. However, the addition of AuNPs can quench the fluorescence intensity of CaS:Ce^3+^@NaYF_4_@lipo-NH_2_ via the FRET process (line b). Initially, the negatively charged surface allows AuNPs to remain stably dispersed in the aqueous solution. Once the sodium chloride (NaCl) is added, the charge balance is disrupted, causing AuNPs to aggregate and leading to the recovery of fluorescence of CaS:Ce^3+^@NaYF_4_@lipo-NH_2_ (line c). In the absence of the KAN, the aptamer attaches to the AuNPs through coordination bonds and enhances the negative electrostatic repulsion of AuNPs, thus enabling them to remain dispersed even in salt solution [48]. Upon the introduction of CaS:Ce^3+^@NaYF_4_@lipo-NH_2_, AuNPs-Apt and CaS:Ce^3+^@NaYF_4_@lipo-NH_2_ electrostatically adsorb together, where the FRET process occurs between them. As a result, the luminescence of CaS:Ce^3+^@NaYF_4_@lipo-NH_2_ is quenched (line d). However, in the presence of KAN, the aptamer specifically binds with the KAN. Without the protection of aptamer, the stability of AuNPs is destroyed in the salt solution, leading to aggregation and a decrease in absorption intensity. This reduces the fluorescence quenching effect on CaS:Ce^3+^@NaYF_4_@lipo-NH_2_ and restores the luminescence of CaS:Ce^3+^@NaYF_4_@lipo-NH_2_ (line e). Therefore, the concentration of KAN in the solution is related to the recovery of fluorescence intensity of CaS:Ce^3+^@NaYF_4_@lipo-NH_2_. To exclude the influence of other factors on the fluorescence intensity of CaS:Ce^3+^@NaYF_4_, we individually incorporated essential substances from the assay system into the CaS:Ce^3+^@NaYF_4_ solution. Subsequently, we assessed its emission spectra, revealing that the introduction of individual substances did not modulate the fluorescence intensity of CaS:Ce^3+^@NaYF_4_ (Fig. S11).

### Optimization of experimental parameters

To establish a detection platform with high sensitivity and detection accuracy, it is important to optimize several key detection parameters. At first, we examined the effect of NaCl concentration on the absorption intensity of AuNPs. The introduction of NaCl to AuNPs solution causes noticeable aggregation of AuNPs (Fig. S12a)_,_ which can be further confirmed by the increase of the hydrodynamic diameter of AuNPs (Fig. S12b) and the change in solution color (Fig. S12c). Besides, with an increase in the NaCl concentration, the absorption intensity of AuNPs also exhibits a gradual decline (Fig. S12d). Upon reaching a concentration of 500 mM, the absorption intensity of AuNPs remains almost unchanged. Thus, the optimal NaCl concentration is determined to be 500 mM. Similarly, the concentration of aptamer, mixture time for NaCl-AuNPs, Apt-AuNPs, and Apt-KAN were also optimized. In short, the following experimental conditions were found to give the satisfying results: (a) the optimal concentration of aptamer is 0.8 μM (Fig. S13a); (b) the mixing time for AuNPs and NaCl is 16 min (Fig. S13b); (c) the optimal incubation time between aptamer and AuNPs is 10 min (Fig. S13c); and (d) the mixing time between KAN and AuNPs is 12 min (Fig. S13d).

### KAN detection

**Fig. 6:**
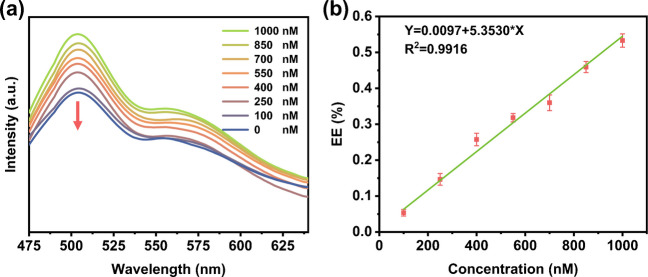
**a** Fluorescence emission spectra (ex@450 nm) of CaS:Ce^3+^@NaYF_4_@lipo-NH_2_ for detecting different concentrations of KAN using the proposed method; **b** Standard curve of the fluorescence enhancement efficiency in different concentration ranges of KAN

Subsequently, the detection of KAN is performed once all the key parameters are optimized. As depicted in Fig. 6a, the emission intensity of CaS:Ce^3+^@NaYF_4_@lipo-NH_2_ at 515 nm exhibits a progressive enhancement with the added KAN concentration. To minimize experimental error, the luminescence spectrum was measured three times at each concentration. The luminescence enhancement efficiency for KAN detection was then established using the formula:

$$\text{Enhancement Efficiency}\left(\text{EE}\right)=\left(F-{F}_{0}\right)/{F}_{0}$$

where *F* represents the luminescence intensity of CaS:Ce^3+^@NaYF_4_@lipo-NH_2_ at 515 nm after the addition of KAN, and *F*_0_​ is the initial luminescence intensity in the absence of KAN. Based on the values of luminescence enhancement efficiency at different concentrations of KAN, a standard curve can be obtained with the formula:

y = 5.3530*x* + 0.0097, where *x* represents the concentration of KAN. The correlation coefficient (*R*^2^) is calculated to be 0.9916, indicating an excellent linear relationship between the luminescence enhancement efficiency and the concentration of KAN **(**Fig. 6b**)**. Meanwhile, the limit of detection (LOD) is determined to be 7.8 nM (S/N = 3). Notably, European Union once established maximum residue limits (MRLs) for kanamycin in various food products, such as 100 μg/kg for egg, 150 μg/kg for milk, 600 μg/kg for liver, etc. [28, 49].Considering the LOD of the as-synthesized sensor is 7.8 nM (~ 3.6 µg/kg), this means that our sensor holds great potential of detecting kanamycin residues in food samples.

In addition, we further compared the proposed method with other recently reported KAN detection methods via different strategies. As presented in Table 1, the linear range and limit of detection in this study were superior to most of the reported results,

**Table 1:** Comparison of the proposed method with recently reported methods for the detection of KAN

Method	Material	Linear range (nM)	LOD (nM)	Reference
Flu^a^ aptasensor	SYBR Green I (SGI)	50 ~ 2 × 10^4^	11.76	[50]
Flu aptasensor	Cu/Ag	80 ~ 1 × 10^3^	13.3	[51]
Flu aptasensor	ZIF-8@TPE^b^	17.16 ~ 17.16 × 10^3^	12.53	[52]
Col aptasensor	Co_3_O_4_	100 ~ 3 × 10^4^	44.2	[53]
Elu aptasensor	g-C_3_N_4_-COOH/ZnSe	1 ~ 1 × 10^5^	0.7982	[54]
Col aptasensor	AuNPs	10 ~ 4 × 10^3^	4	[55]
Col aptasensor	AuNPs	25 ~ 800	20.58	[56]
Flu aptasensor	DNA-AgNCs	100 ~ 2.5 × 10^3^	22.6	[57]
Col aptasensor	OFL-Ti-MN	12.58 ~ 4.6 × 10^5^	15.28	[58]
Flu aptasensor	CaS:Ce^3+^@NaYF_4_@lipo-NH_2_	10 ~ 1 × 10^3^	7.8	This work

^a^Flu, Fluorescent; Col, Colorimetric; Elu, Electrochemiluminescent

^b^ZIF-8, Zeolitic imidazolate framework-8; TPE, tetraacetic acid; OFL-Ti-MN, oxygen-terminated few-layered titanium-based MXene nanosheets

indicating that the proposed aptasensor enables precise detection of KAN. It should be noted that the detection efficiency of the sensor is influenced by several factors, including testing sample, environmental conditions and probe preparation. For example, the complexity of the sample, such as blood, milk, or water, can introduce different interferences that affect sensor performance. Environmental conditions including temperature, humidity, and pH, can also have a significant impact. For instance, temperature fluctuations can change the reaction kinetics between the sensor and the target analyte. Humidity may affect the stability of the sensor, while pH variations may change the charge and solubility of the analyte. Besides, the method of preparing and attaching nanoprobes to the sensor surface also affects the binding efficiency and stability. Variations in the preparation process, such as differences in concentration, coating thickness, or incubation times, can lead to inconsistent sensor responses. Therefore, proper functionalization of the sensor surface ensures stable probe attachment, which is essential for effective detection.

### Specificity of the aptasensor for KAN

**Fig. 7:**
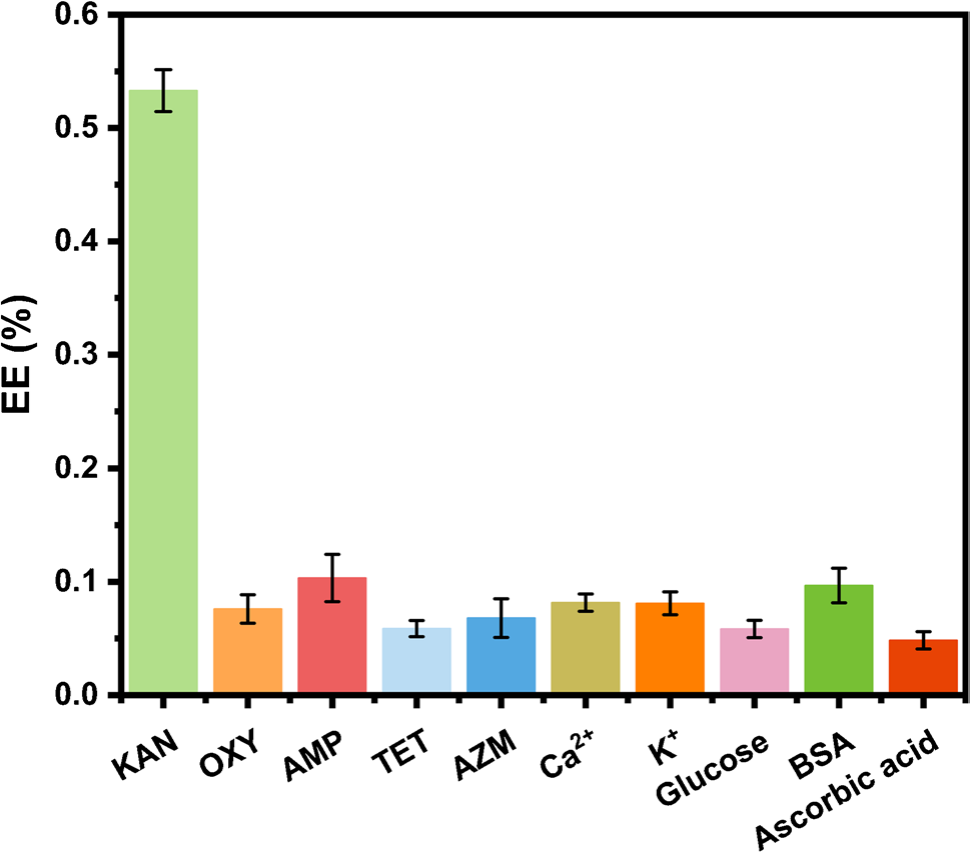
Specificity assessment of the proposed method for KAN against other antibiotics (Oxytetracycline (OXY), Ampicillin (AMP), Tetracycline (TET), and Azithromycin (AZM), and other potential interferences (Ca^2+^, K^+^, glucose, arginine, and ascorbic acid). The enhancement efficiency (EE(%)) in the y-axis indicates the enhancement efficiency of luminescence after addition of different substances

To validate the targeted detection of KAN, four additional antibiotics (Oxytetracycline, Tetracycline, Azithromycin and Ampicillin) and other potential interferences (Ca^2+^, k^+^, glucose, arginine, and ascorbic acid) were used to ensure that the as-developed aptasensor is exclusively sensible with the KAN. As illustrated in Fig. 7, upon adding the analyte at the same concentration, only the introduction of KAN exhibits a luminescence enhancement efficiency exceeding 50%. In contrast, the addition of other antibiotics and substances does not cause a substantial increase in luminescence enhancement efficiency, which indicates a high specificity of the specially designed aptasensor for KAN detection.

### Detection of KAN in real samples

To evaluate the feasibility of this aptasensor for the detection of real samples, tap water and pretreated milk spiked by the different concentrations (50, 100, and 500 nM) of KAN were selected as samples for the recovery assay. As presented in Table 2, the mean recoveries of the spiked KAN samples were between 94.34 and 107.50%, with a relative standard deviation (RSD) range of 1.65–5.80%, indicating that the proposed fluorescent aptasensor is capable of accurate and reproducible detection of KAN in real samples.

**Table 2:** Detection of KAN in spiked tap water and Milk samples

Sample	Spiked (nM)	Detected (nM)	Recovery (%)	RSD (%, *n* = 3)
Tap water	50	47.12 ± 2.13	94.34	4.51
	100	95.15 ± 3.65	95.15	3.83
	500	524.73 ± 8.70	104.95	1.65
Milk	50	53.75 ± 3.12	108.50	5.80
	100	105.26 ± 4.92	105.26	4.67
	500	480.11 ± 9.16	96.02	1.90

## Conclusion

In summary, a core–shell structured CaS:Ce^3+^@NaYF_4_ is developed based on the lattice matching strategy, which exhibits much-enhanced water-resistance capability and luminescence performance than CaS:Ce^3^ bare core. Furthermore, after surface modification with a layer of phosphoric acid bilayer, CaS:Ce^3+^@NaYF_4_ is combined with gold nanoparticles to construct a FRET platform for detecting kanamycin antibiotics. The specially designed aptasensor exhibits excellent sensitivity to kanamycin within the linear range of 100–1000 nM and a detection limit of 7.8 nM. Importantly, the adapsensor has been proven to achieve precise detection of kanamycin in tap water and milk, demonstrating great potential for sensing applications.

## Supplementary Information

Below is the link to the electronic supplementary material.

Supplementary file1 (PDF 156 KB)

Supplementary file2 (PDF 15899 KB)

## Data Availability

The data and materials used in the present study are available from the corresponding authors on reasonable request.
