# Supplementary material for: Lattice matching enables construction of CaS@NaYF4 heterostructure with synergistically enhanced water resistance and luminescence for antibiotic detection
Source: Mikrochim Acta. 2024 Jul 26;191(8):485. doi: 10.1007/s00604-024-06568-x (PMC12779660; doi:10.1007/s00604-024-06568-x)
Supplement: Supplementary file 1 — Supplementary file1 (PDF 156 KB) [file 604_2024_6568_MOESM1_ESM.pdf]

Otto S. Wolfbeis

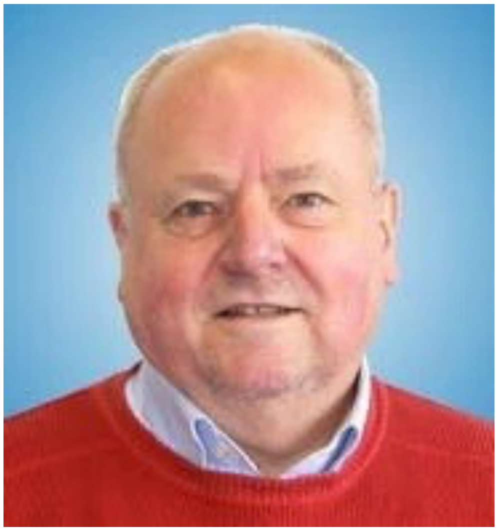

Otto S. Wolfbeis, Professor emeritus at the University of Regensburg, Germany, sadly passed away at age 75 on June 1, 2023. Wolfbeis studied Chemistry at the University of Graz, Austria, and obtained his PhD degree with distinction (1972) under the guidance of Professor H. Junek. From 1972 to 1974, he worked as a postdoctoral fellow with Professor E. A. Koerner von Gustorf at the Max Planck Institute for Radiation Chemistry, Mülheim (Germany). Then, he moved to Technical University of Berlin, where worked with Prof. E. Lippert as a post-doctoral fellow. Wolfbeis began his independent academic career in 1977 when he became an assistant professor in the Department of Chemistry, at Karl-Franzens University, Graz, Austria.

In 1995, Wolfbeis took a full professorship at University of Regensburg, Germany, where he served as the director of Institute of Chemical Sensors and Biosensors. As a prolific researcher, Wolfbeis had published more than 600 scientific papers, mainly in the fields of chemical sensors and biosensors, fluorescent probes and assays, analytical fluorescence spectroscopy, etc. He had also contributed significantly to translating his technologies for commercial use and filed more than 50 patents as a co-inventor, for instance, the test kit for the detection of borderline microalbuminuria (Progen), optical TOC (total organic carbon) test (Merck), Mycobacteria (tuberculosis) growth system and indicator tube (Becton Dickinson), etc. He was a shareholder of the company, Presens GmbH (founded in 1998), which designed and developed optic sensors and imagers for oxygen, pH and CO_2. In 2001, Wolfbeis founded a company Chromeon Gmbh (now part of Active Motif, Carlsbad, California, USA), which manufactured fluorescent labels and materials for ep-genetics research.

Wolfbeis actively contributed to the advancement of scientific publishing in the field of chemistry,, notably through his extensive contributions to various prestigious journals. He founded the Springer Series on Fluorescence (editor from 1999 to 2013), Springer Series on Chemical Sensors and Biosensors (editor from 2000 to 2006), and Bioanalytical Reviews. He served as the editor-in-chief of Microchimica Acta from 2002 to 2019, promoting the journal to become the ultimate platform for cutting-edge research in analytical chemistry. He was also the editorial board member

for many journals, including Sensors & Actuators (1991-2004), Biosensors & Bioelectronics (1994-2000), Angewandte Chemie (2005-2011) and others.

Throughout his career, Wolfbeis supervised over 60 PhD dissertations, 7 Humboldt fellows, and numerous master and diploma works. The impact of Wolfbeis' outstanding work in analytical chemistry cannot be overstated given his numerous contributions to the field. Wolfbeis will always be remembered with respect and gratitude by his students, colleagues and friends for his dedication to Analytical Chemistry and for his generosity and enthusiasm.

Prof. Yong Zhang (City University of Hong Kong)

Prof. Xiaohui Zhu (Shanghai University)
