# Supplementary material for: Lattice matching enables construction of CaS@NaYF4 heterostructure with synergistically enhanced water resistance and luminescence for antibiotic detection
Source: Mikrochim Acta. 2024 Jul 26;191(8):485. doi: 10.1007/s00604-024-06568-x (PMC12779660; doi:10.1007/s00604-024-06568-x)
Supplement: Supplementary file 2 — Supplementary file2 (PDF 15899 KB) [file 604_2024_6568_MOESM2_ESM.pdf]

Supporting information

Lattice matching enables construction of CaS@NaYF$_4$ heterostructure with synergistically enhanced water resistance and luminescence for antibiotic detection

Yao Wang[1,#], Huadong Chen[1,#], Tonghan Zhao[1], Jing Wang[1], Yihan Wu[1], Jinliang Liu[1], Yong Zhang[2*], Xiaohui Zhu[1,*]

1. School of Environmental and Chemical Engineering, Shanghai University, Shanghai, China, 200444

2. Department of Biomedical Engineering, City University of Hong Kong, Hong Kong SAR, 999077, China

[#]Y. Wang and [#]H. Chen contributed equally to this work.

Corresponding Author:

[*]E-mail: yozhang@cityu.edu.hk; xhzhu@shu.edu.cn

Experiment

Chemicals and materials

$Ca(CH_3COO)_2 \cdot H_2O$(99.9%), $Ce(CH3COO)_3 \cdot 4H_2O$(99.99%), Y_2O_3(99.99%), CF_3COO Na(99.99%), CF3COOH, Oleic acid (OA), Oleylamine (OAm), Trioctylamine (TOA), N, N'-diphenylthiourea (DPTU), aminopropyltriethoxysilane (APTES), glucose and a rginine were purch-ased from Sigma-Aldrich (China). 2-distearoyl-sn-glycero-3-phosp hoethanolamine- [(polyethyleneglycol)-2000] (DSPE-PEG2000) phospholipids (lipo) was purchased from Shanghai Ponsure Biotech. Aptamer 5′-TGGGGGTTGAGGCTA AGCCGA-3′ wa-s synthesized by Sangon Biotech (Shanghai) Co., ltd. (Shanghai, Chi na). Kanamycin, Oxytetracycline, Tetracycline, Azithromycin and Ampicillin were pu rchased from Sa-ngon Biotech. sodium chloride (NaCl), potassium chloride (KCl), cal cium chloride ($CaCl_2$), chloroauric acid ($HAuCl_4 \cdot 3H_2O$) and sodium citrate were obtai ned from Sinopharm Chemical Reagent Co., Ltd. (Shanghai, China)

Apparatus

The transmission electron microscopy (TEM) images were obtained by an HT7800 (Hitachi, Japan) transmission electron microscope at an acceleration voltage of 100 kV. X-ray diffraction (XRD) pattern was performed on a 7000S/L X-ray powder diffractometer (Shimadzu, Japan). Fluorescence excitation and emission spectra were recorded by an FS5 fluorescence spectrophotometer (Edinburgh, UK) with an excitation slit of 5 nm and an emission slit of 5 nm. Zeta potential and dynamic light scattering (DLS) were recorded on the Malvern Zetasizer Nano ZEN3700 (Malvern, USA). The mean hydrodynamic diameter and the mean zeta potential with the corresponding error as standard deviations from three measurements were determined. Fourier transform infrared spectra (FTIR) were recorded in KBr discs on a Nicolet iS10 FTIR spectrometer (Thermo Fisher, USA).

Synthesis of $CaS:Ce^{3+}$ nanoparticles

A certain amount of $Ce(CH_3COO)_3$ solution (0.01M) was added to a three-necked flask, and the temperature was set to 130°C to evaporate the water content. Subsequently, 0.5 mmol of $Ca(CH_3COO)_2$, 1 mL of oleic acid, 6 mL of oleylamine, and 3 mL of trioctylamine were added. The resulting mixed solution was heated in a vacuum to 120°C for 30 minutes and then further heated to 160°C for another 30 minutes. The solution turned transparent and clear after $Ca(CH_3COO)_2$ completely dissolved. The heating was then

turned off, allowing the solution to naturally cool to below 80°C. Subsequently, 2 mmol of N,N'-diphenylthiourea was dissolved in 10 ml of methanol and sonicated for 10 minutes. This solution was added to the previous mixture, and the heating was turned on to raise the temperature to 110°C, maintaining it for 30 minutes to remove the methanol. Subsequently, a vacuum was applied for 15 minutes, and the reaction was carried out in an argon atmosphere at 320°C for 1 hour. After the reaction was completed, the solution was naturally cooled to room temperature, and ethanol was added. The product was centrifuged and washed multiple times with ethanol and cyclohexane, and the resulting product was dissolved in 5 ml of cyclohexane.

Synthesis of $Y(CF_3COO)_3$

Take 5 mmol of Y_2O_3 and add it to a round-bottom flask. Then, after adding 30 mL of deionized water to the flask, slowly introduce 6 mL of CF_3COOH. Ensure thorough mixing of the solution, and transfer it to an oil bath at 90°C for refluxing for approximately 12 hours. Once the solution becomes clear and transparent, remove it from the reflux apparatus, and heat it to 120°C to evaporate the solution. Finally, dry the obtained product in an 80°C oven for several hours.

Synthesis of $CaS:Ce^{3+}$@$NaYF_4$ core-shell nanoparticles

Take 0.25 mmol of $Y(CF_3COO)_3$ and 0.25 mmol of CF_3COONa and introduce them into a three-necked flask. Subsequently, inject 10 mL of oleylamine into the flask. Following thorough mixing of the solution, heat it to 160°C and maintain this temperature for 1 hour until the trifluoroacetates are completely dissolved. Turn off the heat source and allow the solution to naturally cool to below 80°C. Take 2.5 mL of the synthesized $CaS:Ce^{3+}$ cores and add it to the above solution. Heat the solution once more to 160°C and maintain it for 30 minutes to eliminate cyclohexane. Then, under an argon atmosphere, subject the solution to vacuum treatment for 15 minutes and heat it to 220°C, maintaining this temperature for 2 hours. Turn off the heat source, permitting the solution to naturally cool to room temperature. Add ethanol to the solution and conduct centrifugation. Wash the precipitate multiple times with ethanol and cyclohexane. Finally, dissolve the obtained product in 5 mL of cyclohexane. By adjusting the amounts of trifluoroacetic yttrium and trifluoroacetic sodium added, $CaS:Ce^{3+}$@$NaYF_4$ with varying shell thicknesses were synthesized.

Synthesis of lipo-coated $CaS:Ce^{3+}@NaYF_4$ nanoparticles

We performed surface modification on $CaS:Ce^{3+}@NaYF_4$ using a method previously reported[1]. Specifically, 10 mg of oleylamine-capped $CaS:Ce^{3+}@NaYF_4$ nanoparticles were dispersed in a chloroform solution (5 mL) containing 30 mg of DSPE-PEG2000 phospholipids, and the mixture was sonicated for 2 minutes. Subsequently, the mixture was subjected to vacuum drying at 40°C using a rotary evaporator, forming a lipid film on the inner wall of the flask. Then, the lipid film was hydrated with distilled water (5 mL) and dissolved after vigorous sonication for 5 minutes. The aqueous solution was centrifuged at 10000 rpm for 10 minutes to remove excess lipids.

Synthesis of $CaS:Ce^{3+}@NaYF_4@lipo\text{-}NH_2$ nanoparticles

To change the surface charge of $CaS:Ce^{3+}@NaYF_4@lipo$ nanoparticles, we redissolved them in 10 mL of chloroform and added 200 μL of APTES to the solution, which was subsequently transferred to an oil bath and refluxed for 12 h at 80 °C. After centrifugation, the precipitate was washed several times with chloroform and distilled water and finally redispersed in distilled water for subsequent use.

Synthesis of AuNPs

First, a condensation reflux unit was set up in an oil bath. 0.25 mL of 0.1 mol/L $HAuCl_4$ and 100 mL of ultrapure water were mixed and added to a 250 mL two-neck flask. The mixture was heated and stirred at 130 °C to bring the solution to a vigorous boil. A proportion of 1% (wt%) trisodium citrate solution was quickly added and stirred at high speed. After the solution in the flask changed color, boiling reflux was continued for 20 minutes, then stirring was maintained and cooled to room temperature. Large particles were filtered out using a 0.22 μm filter membrane, then transferred the AuNPs to a clean glass vial and stored in a refrigerator at 4 °C under light protection for later use. AuNPs with different sizes were successfully synthesized by adding different amounts of sodium citrate solution.

The procedure of KAN detection

The procedure for kanamycin detection under the optimized conditions is as follows: Initially, 100 μL of KAN solution with varying concentrations was introduced to 50 μL of aptamer solution (1.0 μM). The resultant mixture was thoroughly shaken and allowed to incubate for 12 minutes. Afterward, 150 μL of AuNPs (7.6 nM) was added and

reacted for another 10 min. Following this, 100 μL of NaCl solution (500 nM) was added and thoroughly shaken for 16 min. Finally, 200 μL of $CaS:Ce^{3+}@NaYF_4@lipo-NH_2$ (1mg/mL) was introduced to the system. Upon thorough mixing of the solution, the emission spectrum was promptly measured in the 475-675 nm range using a fluorescence spectrometer.

Detection of KAN in real samples

The tap water from the laboratory (Shanghai University) and the milk obtained from the local supermarket were used for real sample analysis. The samples were pretreated according to the previous report[2]. First, 500 μL of HAc (20% v/v) was added to 3.0 mL of pure milk, and the mixture was stirred thoroughly to completely precipitate the proteins in the milk. The mixture was then centrifuged at 10,000 rpm/min for 15 min. The supernatant was filtered through a 0.22 μm filter membrane. The filtrate was then diluted 10 times with Tris–HCl buffer (pH 7.4) and stored at 4 °C for further use. Subsequently, different concentrations of kanamycin were spiked into the milk and tap water. Next, the proposed method was used to detect KAN in the spiked milk and tap water. Three parallel measurements were performed for each sample.

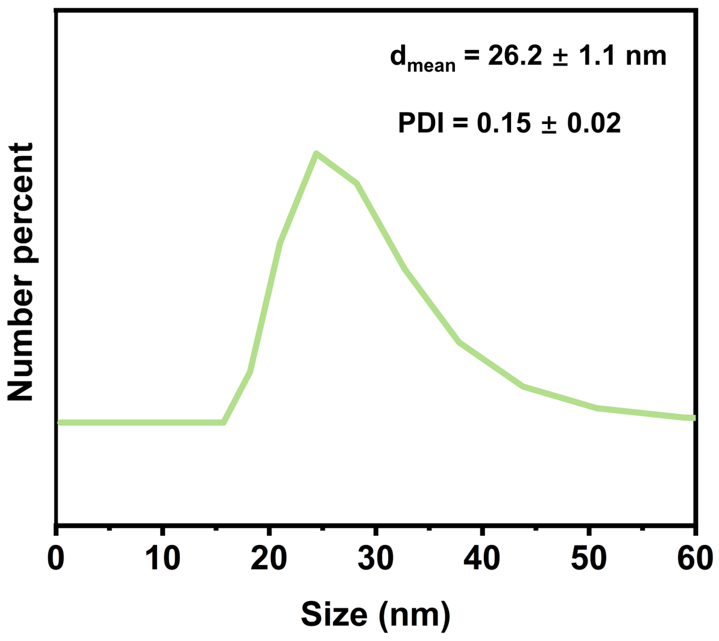

Figure S1. Hydrodynamic diameter distribution of CaS:Ce^{3+} nanoparticles.

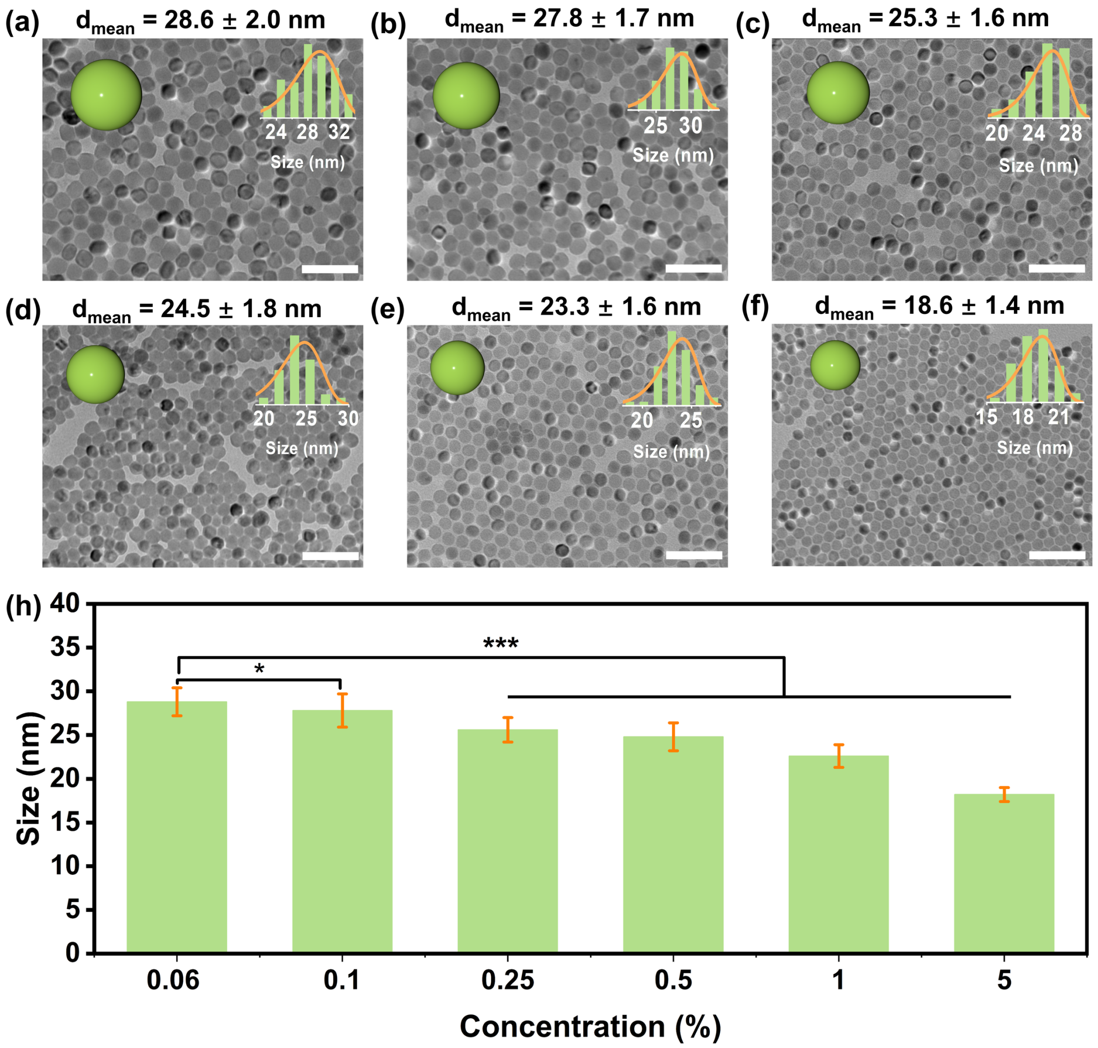

Figure S2. (a-f) TEM images and corresponding size distribution of the CaS:x% Ce^{3+}(x=0.06, 0.1, 0.25, 0.5, 1, 5) nanoparticles. The scale bar is 100 nm. (g) Statistical analysis of the particle size of CaS:x% Ce^{3+} nanoparticles. $*p < 0.05$; $***p < 0.001$.

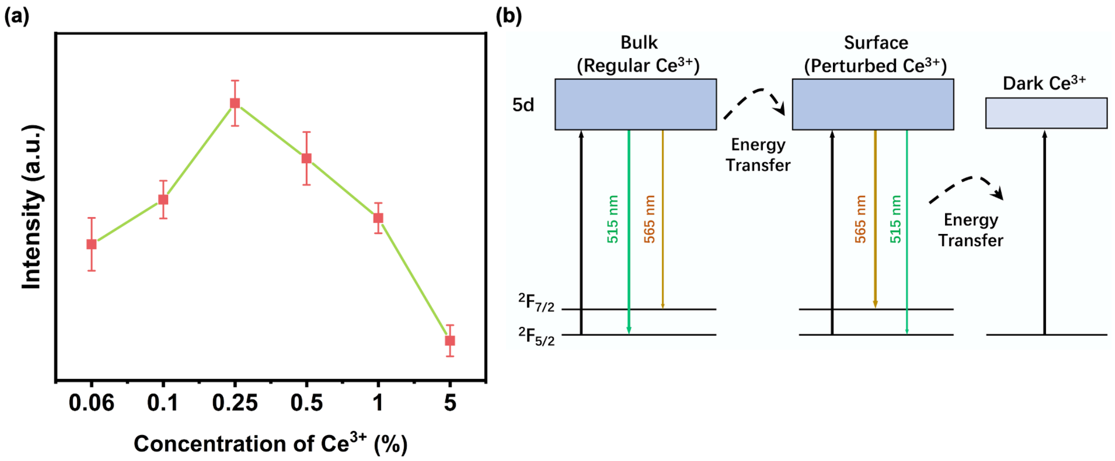

Figure S3. (a). Emission intensity at 515 nm for CaS:x% Ce^{3+} nanoparticles doped with different Ce^{3+} concentrations; (b). Schematic of the luminescence processes in CaS:Ce^{3+} nanoparticles.

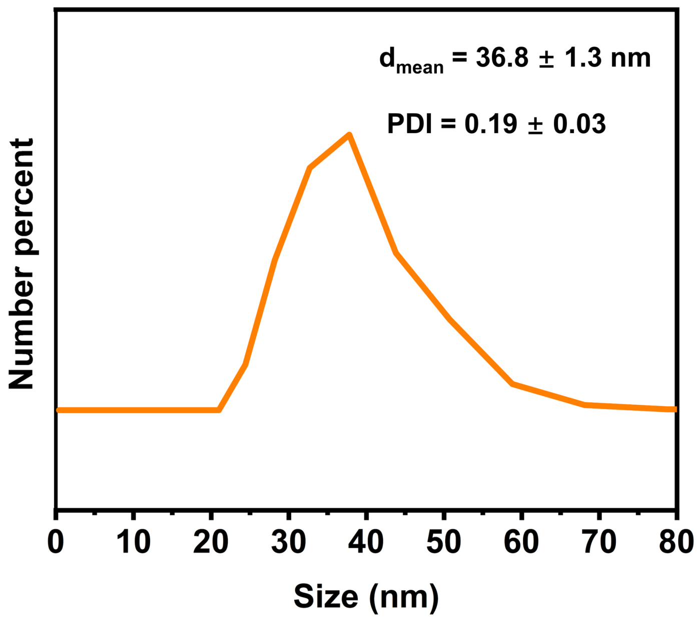

Figure S4. Hydrodynamic diameter distribution of $CaS:Ce^{3+}@NaYF_4$ nanoparticles.

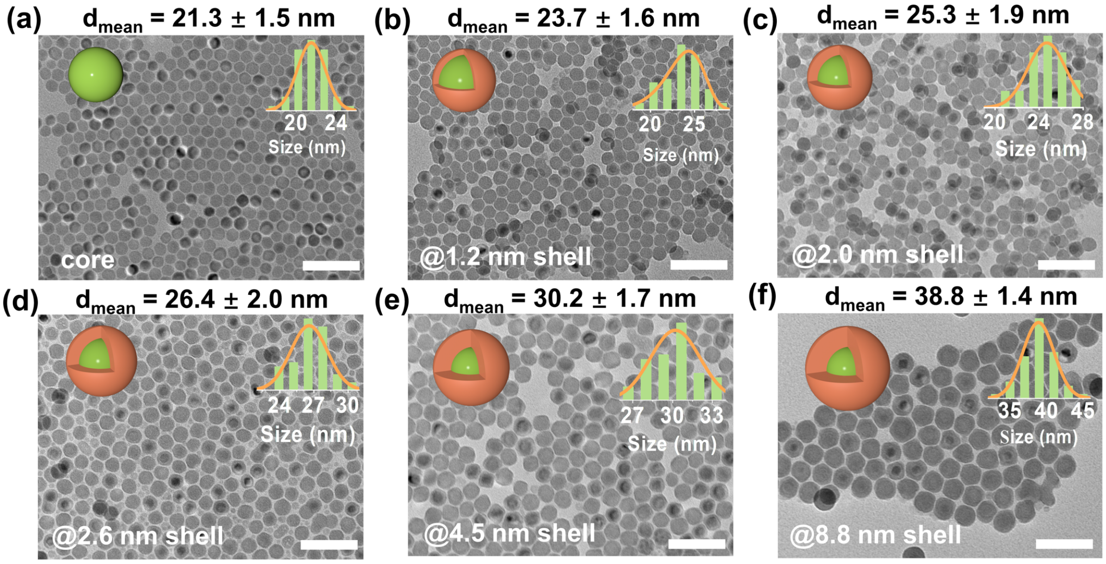

Figure S5. (a) TEM image and corresponding size distribution of the CaS:Ce^{3+} nano-particles; (b-f) TEM images and the corresponding size histograms of the CaS: Ce^{3+}@$NaYF_4$ nanoparticles with different shell thicknesses synthesized from (a) as seeds. The scale bar is 100 nm.

Table S1. Photoluminescence quantum yield (PLQY) of $CaS:Ce^{3+}$, $CaS:Ce^{3+}$@$NaYF_4$ and $CaS:Ce^{3+}$@$NaYF_4$@lipo nanoparticles.

Samples	$CaS:Ce^{3+}$	$CaS:Ce^{3+}$@$NaYF_4$	$CaS:Ce^{3+}$@$NaYF_4$@lipo
PLQY	31.4%	40.8%	43.6%

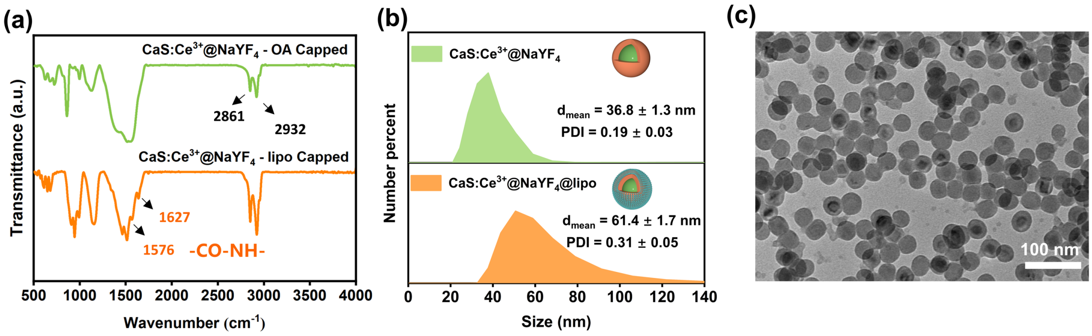

Figure S6. (a) FTIR spectra of the OA-capped CaS:Ce^{3+}@$NaYF_4$ and the lipo-coated CaS:Ce^{3+}@$NaYF_4$; (b) Hydrodynamic diameter distribution of CaS:Ce^{3+}@$NaYF_4$ and CaS:Ce^{3+}@$NaYF_4$@lipo nanoparticles; (c) TEM of CaS:Ce^{3+}@$NaYF_4$@lipo nanoparticles.

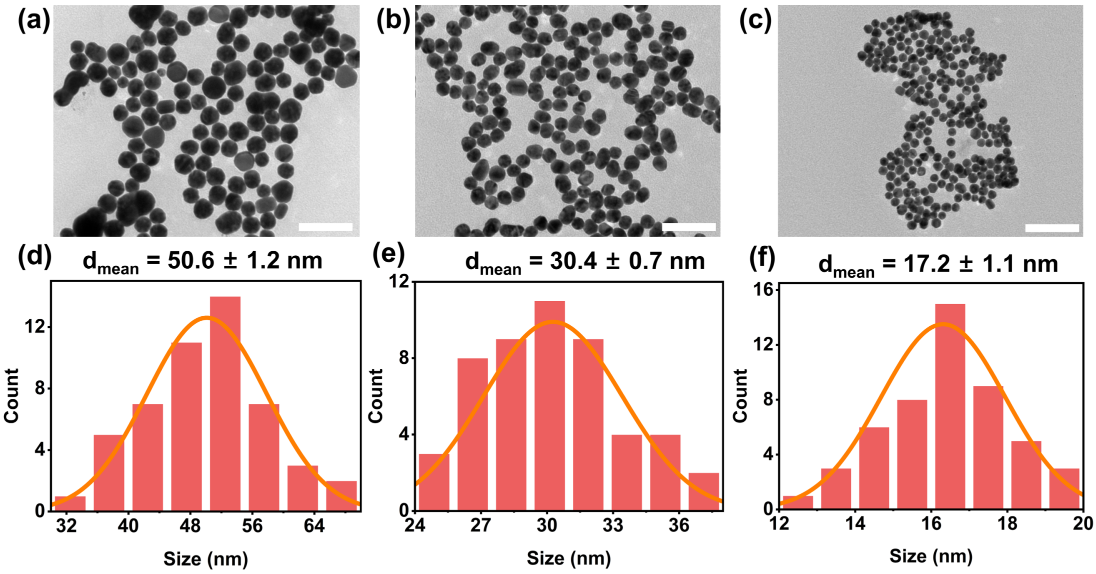

Figure S7. (a-c) TEM images of AuNPs synthesized by adding different amounts of sodium citrate (from left to right: 1.0 mL, 1.5 mL, and 2.0 mL) and the corresponding size distribution (d-f). The scale bar is 100 nm.

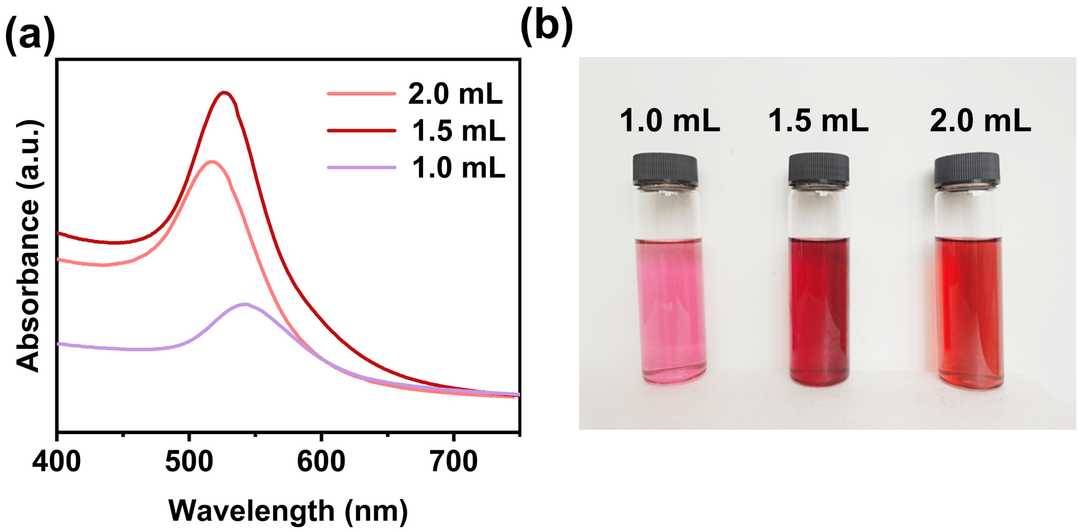

Figure S8. (a) UV-Vis absorption spectra of AuNPs solution synthesized by adding different amounts of sodium citrate and corresponding Photograph in water solution (b).

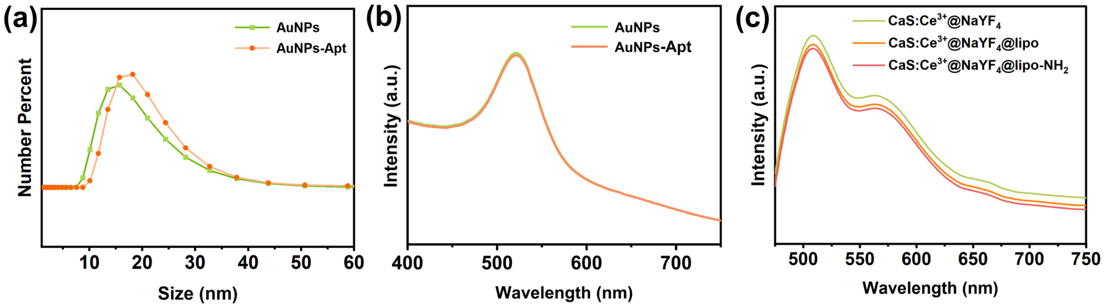

Figure S9. (a) Hydrodynamic diameter distribution of AuNPs before and after binding with aptamer, and corresponding UV-Vis absorption spectra (b); (c) Fluorescence emission spectra (ex@450 nm) of CaS:Ce^{3+}@$NaYF_4$, CaS:Ce^{3+}@$NaYF_4$@lipo and CaS:Ce^{3+}@$NaYF_4$@lipo-NH_2 nanoparticles.

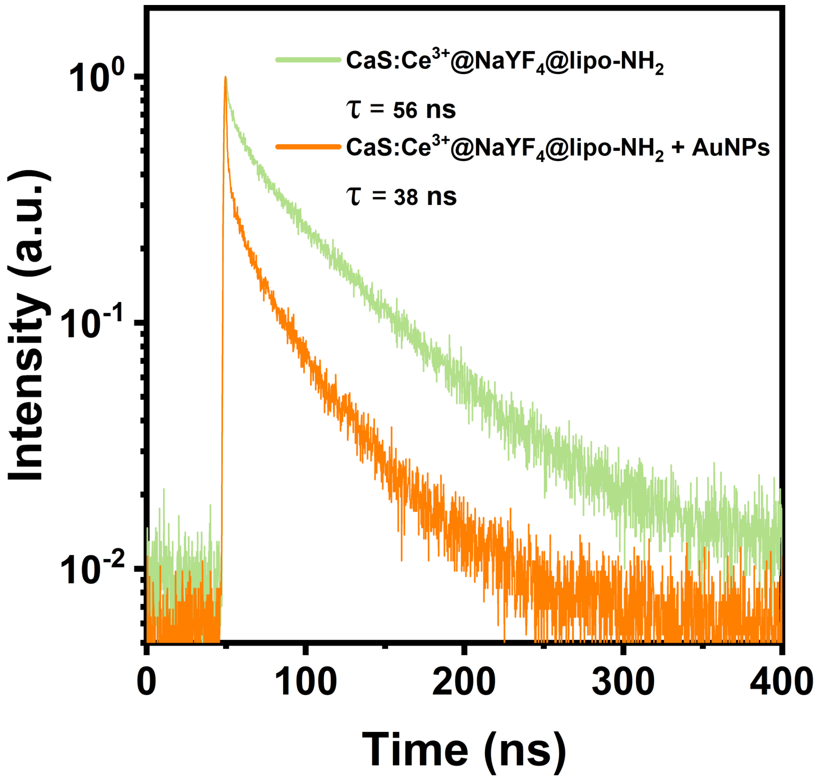

Figure S10. Fluorescence decay curves of $CaS:Ce^{3+}@NaYF_4@lipo\text{-}NH_2$ nanoparticles and $CaS:Ce^{3+}@NaYF_4@lipo\text{-}NH_2$ + AuNPs systems emitted at 515 nm under the excitation of 450 nm.

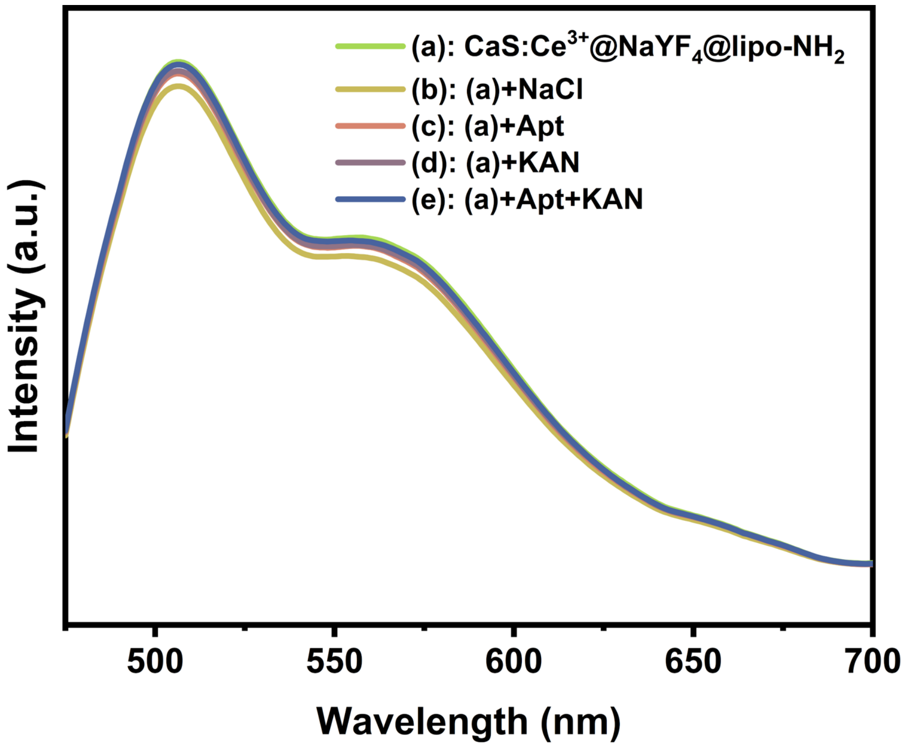

Figure S11. Fluorescence emission spectra (ex@450 nm) of CaS:Ce^{3+}@$NaYF_4$@lipo-NH_2 nanoparticles with various components.

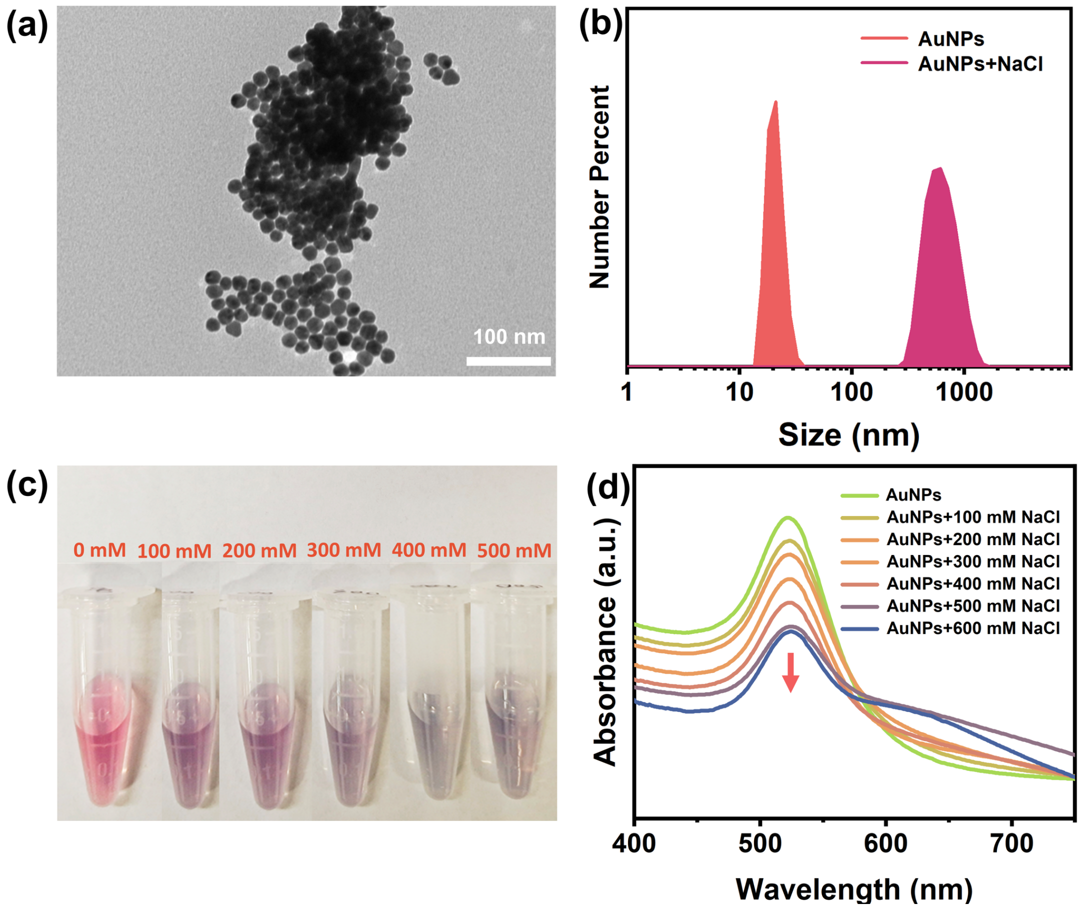

Figure S12. (a) TEM image of AuNPs aggregation caused by addition of NaCl; (b) Hydrodynamic diameter distribution of AuNPs and AuNPs dissolved in a salt solution; (c) Color change of AuNPs solution after addition of different concentrations of NaCl and corresponding UV-Vis absorption spectra (d).

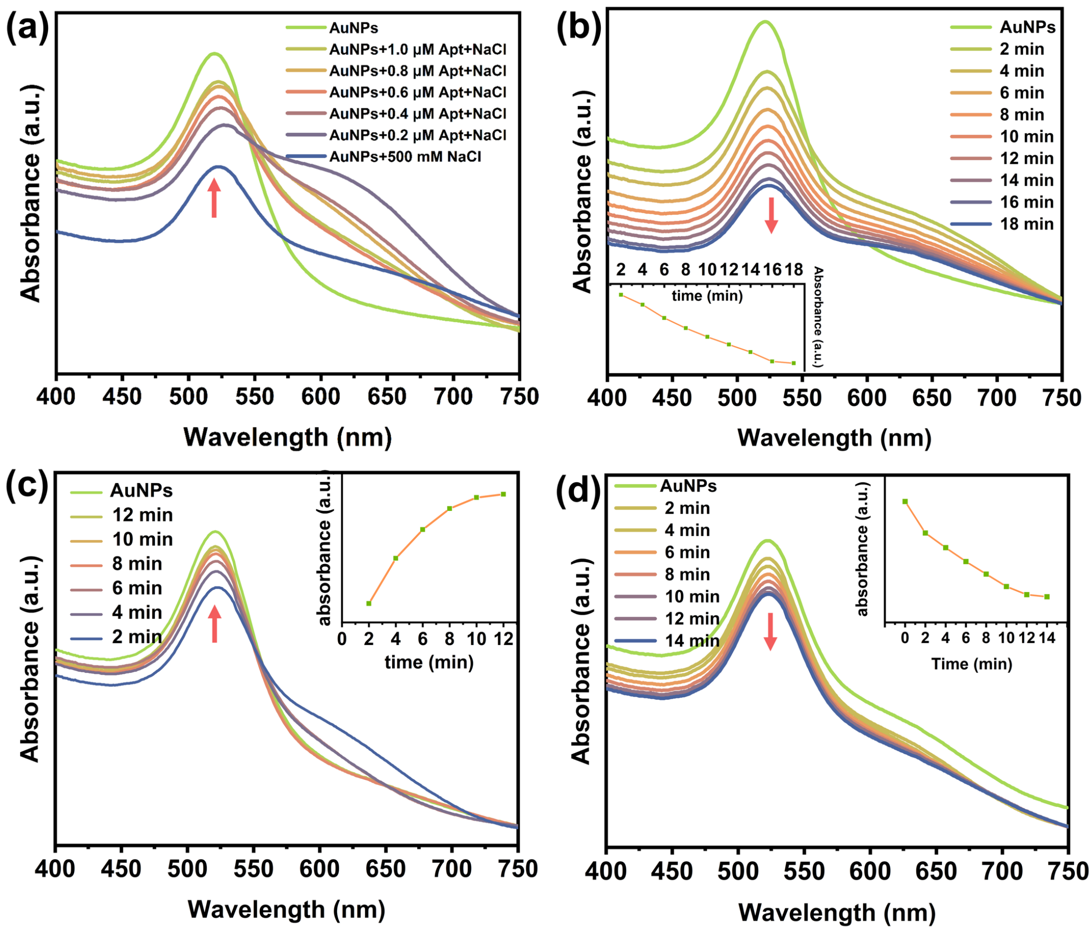

Figure S13. (a) UV-Vis absorption spectra of AuNPs measured by addition of different concentrations of aptamer and 500 mM NaCl; (b) Time-dependent UV-Vis absorption spectra of AuNPs in salt solution; (c) Time-dependent UV-Vis absorption spectra of AuNPs-Apt in salt solution; (d) Time-dependent UV-Vis absorption spectra of AuNPs-Apt-KAN in salt solution.

References

1. Zhang M, Zheng W, Liu Y, et al (2019) A New Class of Blue-LED-Excitable NIR-II Luminescent Nanoprobes Based on Lanthanide-Doped CaS Nanoparticles. Angew Chem Int Ed 58:9556–9560. https://doi.org/10.1002/anie.201905040
2. Chen Y, Zhao L, Wu X, et al (2022) Spontaneously formation of peroxidase mimetics on $CuWO_4$ for homogeneous and universal aptasensing platform. Sensors and Actuators B: Chemical 367:132040. https://doi.org/10.1016/j.snb.2022.13204
